# Deep learning‐based synthetic CT generation for MR‐only radiotherapy of prostate cancer patients with 0.35T MRI linear accelerator

**DOI:** 10.1002/acm2.13327

**Published:** 2021-06-28

**Authors:** Reza Farjam, Himanshu Nagar, Xi Kathy Zhou, David Ouellette, Silvia Chiara Formenti, J. Keith DeWyngaert

**Affiliations:** ^1^ Department of Radiation Oncology Weill Cornell Medical College New York New York USA; ^2^ Public Health Science Weill Cornell Medical College New York New York USA

**Keywords:** deep learning, synthetic CT, 0.35 T MRI‐Linac

## Abstract

**Purpose:**

To develop a deep learning model to generate synthetic CT for MR‐only radiotherapy of prostate cancer patients treated with 0.35 T MRI linear accelerator.

**Materials and Methods:**

A U‐NET convolutional neural network was developed to translate 0.35 T TRUFI MRI into electron density map using a novel cost function equalizing the contribution of various tissue types including fat, muscle, bone, and background air in training. The impact of training time, dataset size, image standardization, and data augmentation approaches was also quantified. Mean absolute error (MAE) between synthetic and planning CTs was calculated to measure the goodness of the model.

**Results:**

With 20 patients in training, our U‐NET model has the potential to generate synthetic CT with a MAE of about 29.68 ± 4.41, 16.34 ± 2.67, 23.36 ± 2.85, and 105.90 ± 22.80 HU over the entire body, fat, muscle, and bone tissues, respectively. As expected, we found that the number of patients used for training and MAE are nonlinearly correlated. Data augmentation and our proposed loss function were effective to improve MAE by ~9% and ~18% in bony voxels, respectively. Increasing the training time and image standardization did not improve the accuracy of the model.

**Conclusion:**

A U‐NET model has been developed and tested numerically to generate synthetic CT from 0.35T TRUFI MRI for MR‐only radiotherapy of prostate cancer patients. Dosimetric evaluation using a large and independent dataset warrants the validity of the proposed model and the actual number of patients needed for the safe usage of the model in routine clinical workflow.

## 1 INTRODUCTION

MRI linear accelerator (MRI‐Linac) is increasingly used for adaptive planning and target motion tracking in radiotherapy of prostate cancer.^1^ In our institution, prostate cancer patients comprise ~67% of all patients treated with MRI‐Linac. In 0.35T MRgRT (ViewRay, Inc. OH, USA) workflow, each patient is simulated with both CT and MRI linear accelerator. MRI simulator images are used for target delineation and organs at risk (OARs) contouring and CT scans are used for dose calculation after it is registered onto MRI simulation image. Acquiring two scans in MRgRT increases the time, cost, and complexity of the simulation procedure and may also impose mis‐registration issue. The ability to derive electron density information directly from therapeutic MRI could resolve these issues and is an urgent need and an active area of research in radiation oncology.

Numerous approaches have been proposed so far to estimate electron density map (synthetic CT) for MR‐only radiotherapy of pelvis anatomy.^2^, ^3^, ^4^, ^5^, ^6^, ^7^, ^8^, ^9^, ^10^ Tissue classification with bulk density assignment,^2^, ^3^, ^4^, ^5^ atlas‐based^6^, ^7^ and deep‐learning^8^, ^9^, ^10^ approaches are some of the main categories. Mis‐classification of air and bone and loss of within‐cluster details are the primary shortcomings of the clustering techniques. Also, atlas‐based approaches rely on the accuracy of the deformable image registration of an atlas with known electron density map to a sample patient. Hence, they suffer from insufficient precision due to inherent registration errors caused by both intermodality and interpatient anatomical variations. Also, it may take time to generate the final product making them inappropriate for a busy clinic. In contrast, deep learning techniques have shown promising results in terms of both accuracy and clinical efficiency.^8^, ^9^, ^10^ While the training step can be done offline with substantial amount of data, generation of synthetic CT takes less than a minute making this approach a suitable tool to generate synthetic CT for clinical use.

Most of the deep learning approaches presented in the literature for synthetic CT generation has been focused on the head and neck anatomy^11^, ^12^, ^13^, ^14^, ^15^, ^16^, ^17^ and less studies have been conducted on the pelvis region.^8^, ^9^, ^10^ In most of these works, U‐NET was the main deep learning architecture used in the model development step. However, there are some works using ResNet^17^ as the base convolutional neural network (CNN). General adversarial networks (GAN) have been also utilized^10^, ^13^, ^14^, ^15^, ^16^, ^17^ to enhance the performance of the U‐NET architecture. In GAN techniques, two CNN models, named generator and discriminator, are simultaneously trained wherein the generator is the predictive architecture such as U‐NET while discriminator is a simpler network whose role is to distinguish between the synthetic (fake) and planning (real) CT. The ultimate goal of the network is to train a model generating a synthetic CT that cannot be distinguished from real CT. In addition, cycle GAN models have also been used^14^, ^16^ to eliminate the misalignment issue exist between the coregistered CT and MRI. In cycle GAN approaches, two CNN models are trained consecutively. The output of the first model is used to generate the synthetic CT which will be then used as an input for the second model which is supposed to map the synthetic CT to the original MRI image. Nonetheless, almost all of these approaches have focused on the utility of the model and little has been done on the impact of image preprocessing, dataset size, and training variables. Most of these works was also developed with high quality MRI images and only a few attempts are available for 0.35T MRI data.^10^ In this work, we developed a U‐NET convolutional neural network to derive electron density map from 0.35T MRI data. We also proposed a novel cost function to improve the accuracy of the model in estimating the electron density value in bony regions. We also quantified the impact of data augmentation, dataset size and some image preprocessing techniques.

## 2 MATERIALS AND METHODS

### 2.1 Dataset

Thirty patients diagnosed with prostate cancer and treated with 0.35T MRI‐Linac were randomly selected for this IRB‐approved study. The patients’ age ranged from 54 to 86 with a median of 74. Twenty four patients had intact prostate and underwent stereotactic body radiotherapy. The rest of the patients were treated with conventional fractionation. No patient had metal implant in his pelvic region. No other exclusion criteria existed to form the dataset.

**FIGURE 1:**
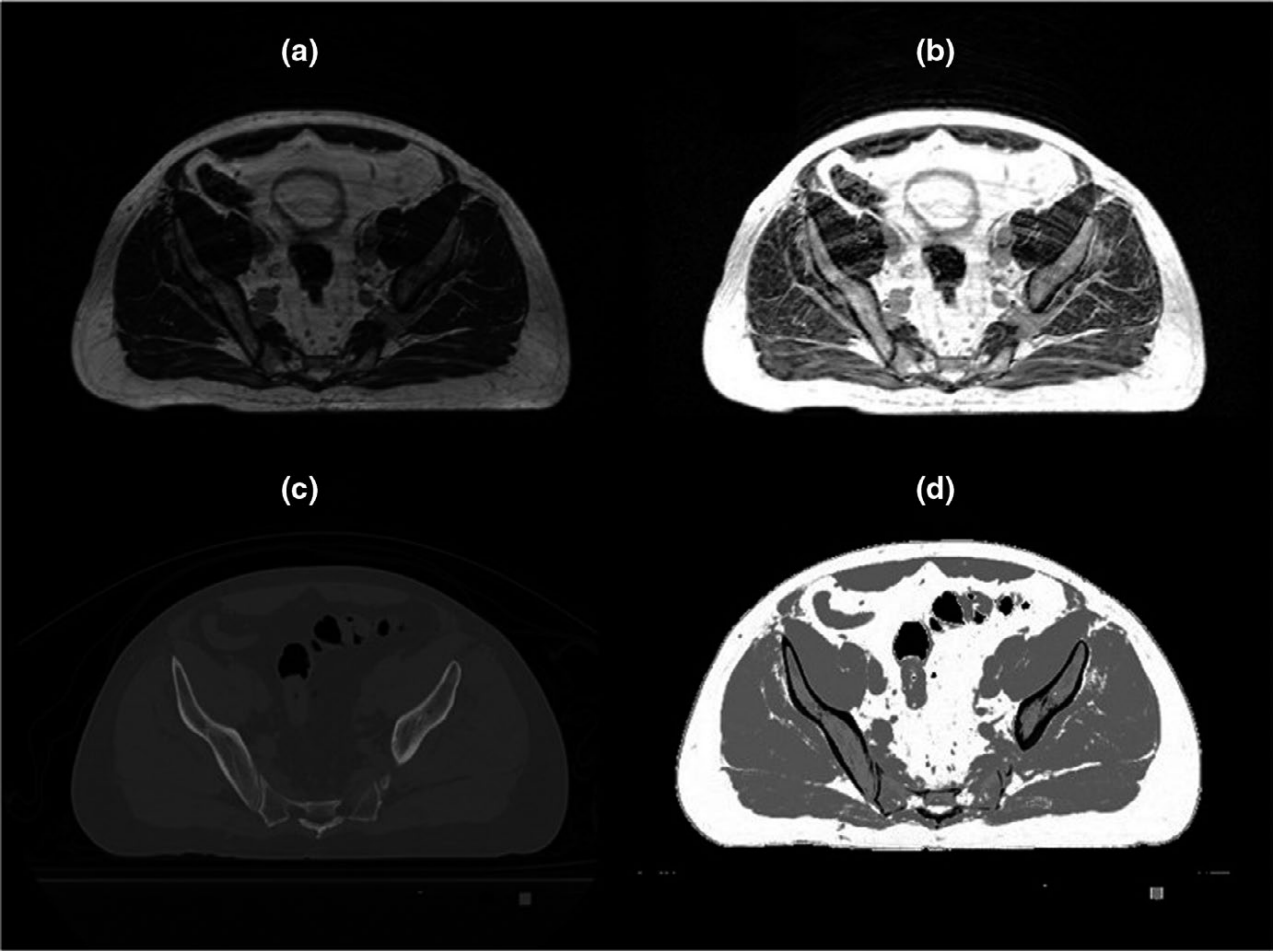
Image pre‐processing steps performed prior to deforming planning CT to TRUFI MRI image. (a) original TRUFI MRI, (b) standardized TRUFI MRI, (c) planning CT, and (d) intensity‐matched CT

In 0.35 T MRgRT workflow, each patient is initially simulated with both CT and MRI‐Linac. MRI scans were acquired using true fast imaging with steady‐state precession (TRUFI) pulse sequence with flip angle of 60°, TR/TE = 3.37/1.45 ms, in‐plane resolution and slice thickness of 1.5 mm and matrix size of 300 ∗ 334, Figure 1a. MRI scans were acquired using a two‐part 12‐element phase array torso receive coils placed below and above the patient. Each half (upper and lower) of the coil had 6 elements and could function independently. CT scans were acquired using SIEMENS scanner with 120 KVP, in‐plane resolution of 1 mm × 1 mm, slice thick of 2 mm and matrix size of 512 × 512, Figure 1c. MRI simulator images are used for target delineation and OARs contouring and CT scans are used for dose calculation.

### 2.2 MRI‐CT atlas creation

The methodology introduced in Farjam et al.^7^ was adjusted and used in this work to create MRI‐CT atlas. A brief description of each step is explained in the following subsections.

#### 2.2.1 MRI: Intensity inhomogeneity correction and image standardization

An image processing approach^18^ was initially used to reduce the intensity inhomogeneity of MR images due to field nonuniformity and tissue susceptibility effects. To reduce the scanner‐dependent intensity variation, a landmark‐based standardization technique was then used to standardize the MR intensity histogram (TRUFI_stdn_). To derive tissue‐specific landmarks, an area in fat and muscle was manually contoured for each MRI scan. The median intensity of these regions was utilized as a landmark corresponding to each tissue. The intensity value corresponding to each landmark was then transferred to a fixed value, 400 for muscle and 800 for fat, in the newly constructed intensity map. Air intensity was also set to zero in the newly constructed map. Intensity values between air and muscle, between muscle and fat, and above the fat landmark were also linearly transferred, Figure 1b. This procedure was applied to each patient independently.

#### 2.2.2 CT: Intensity matching

The intensity matching approach introduced in Farjam et al.^7^ was used to enhance the similarity between CT and MRI scans to facilitate the image registration between CT and MR images for atlas formation. Using thresholding (HU < −250), air voxels were initially excluded from CT image. Fuzzy‐c‐means (FCM) clustering was then used to classify the remaining voxels into fat, muscle, and bone regions. FCM assigns fuzzy membership to each data point corresponding to each cluster center based upon the distance between the cluster center and the data point.^19^ Bone voxels were replaced by air intensity as described previously^7^ to match the intensity of bony region in MR images. The new CT (CT_bs_) was then standardized (CT_bs,stnd_) such that fat and muscle cluster centers in CT_bs_ matched the intensity of the fat and muscle landmarks in the standardized TRUFI MRI, Figure 1d.

#### 2.2.3 Image registration

To create MRI‐CT atlas, rigid registration followed by B‐spline deformable registration was used in Plastimatch^20^ to deform CT_bs,stnd_ onto corresponding TRUFI_stdn_. A sampling rate of 1 × 1 × 1 was used to avoid smoothing and blurring effects. In deformation step, mean square error was utilized as cost function to fine‐tune the rigidly aligned images. The resulting deformation matrix was then applied to the planning CT to obtain MRI‐CT atlas (TRUFI, CT_reg_). This procedure was performed for all 30 patients. All calculation was done in MATLAB^21^ (Version 9.7, R2020a, MathWorks, Inc.).

### 2.3 Model training

#### 2.3.1 Network architecture

**FIGURE 2:**
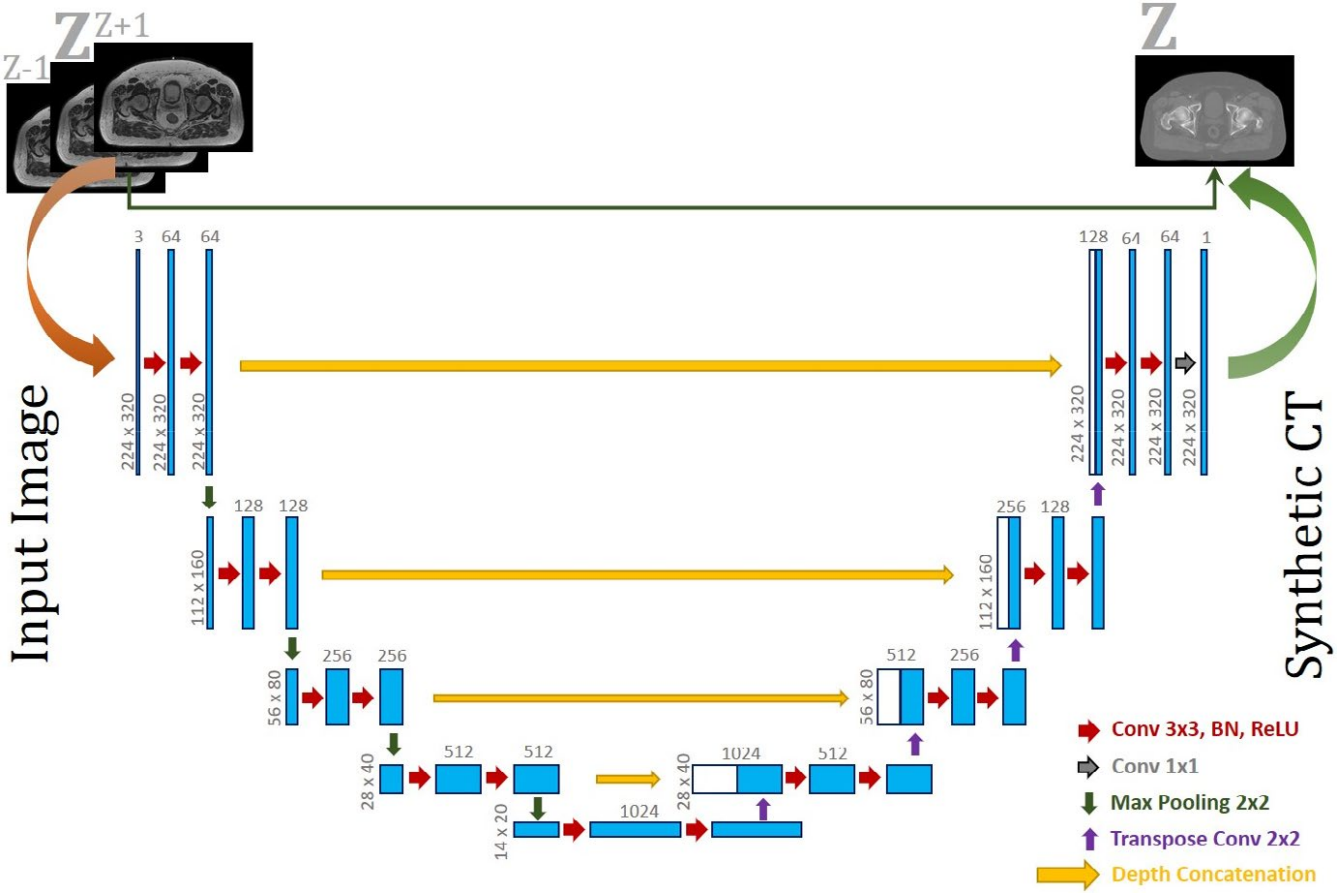
Architecture of the UNET model used in this study to convert 0.35T TRUFI image to electron density map. The model takes three‐channel TRUFI MRI image and returns a synthetic CT corresponding to the middle channel. BN, batch normalization

A U‐NET architecture^11^ was developed in this work to translate TRUFI MRI scans into corresponding electron density map. The U‐NET model consists of contracting (encoding) and expanding (decoding) paths with skip connections between the corresponding encoder and decoder to provide local information to the global information while up‐sampling. Encoding path comprises of blocks of two convolutional layers followed by a max pooling layer that squeezes the input image and increases the number of filters by a factor of two. Each convolution layer is followed by a Batch normalization^22^ and a ReLU^23^ activation layer. Decoding path includes one transposed convolution to perform up‐sampling followed by two convolutional layers as described earlier. The network input is a three‐channel image with a size of 224 × 320 (explained in the following section) and the output is a one‐channel image with the same size of the input image. U‐NET was chosen due to its popularity and lower complexity. Figure 2 shows the architecture of the U‐NET model used in this study along with its input and output images.

#### 2.3.2 Network input

For each MRI scan, body surface was automatically contoured using ViewRay treatment planning system and exported to MATLAB as a structure DICOM file. The center of mass, *M*_body_, in body contour was calculated and used to translate the co‐registered MRI‐CT pair in a way that *M*_body_ overlays the image center. The most posterior voxel of the body contour, *P*_body_, was also identified and the centered MRI‐CT was translated again so that *P*_body_ is 10 voxels away from the image horizontal edge. All voxels outside of the body contour was considered as background air and set to zero. To expedite the training process and save memory, background margin in MRI‐CT atlas was truncated in a way that each MR‐CT pair has an in‐plane matrix size of 224 × 320. That was the minimum matrix size encompassing body contours from all patients and allowing at least four times of max pooling needed to train the model. A three‐channel input image, MRI_in,Z_, was then constructed for each three consecutive 2‐dimensional (2D) MRI slices (Z − 1, Z, Z + 1) where Z denotes the slice number. For the most superior and inferior slice, the second from the last slice was used for channels one and three.

#### 2.3.3 Loss function: Tissue‐specific weighting

Our U‐NET model takes MRI_in,Z_ as input and returns synthetic CT (sCT) corresponding to the middle channel as output. The training process is carried out by updating the model's trainable parameters, θ, through iterations. In each iteration, the loss function which is an average voxel‐wise difference between the output image (sCT*_θ_*_,Z_) and the ground truth, co‐registered CT corresponding to middle channel, CT_reg, Z_, is calculated and used to update the model parameters:

$${U‐NET}_{\theta }\left({\mathrm{MRI}}_{in,Z}\right)\to {\mathrm{sCT}}_{\theta ,Z}$$

(1) $$L\left(\theta \right)=\frac{1}{N*B}\sum_{p=1}^{N}\sum_{k=1}^{B}\left|{\mathrm{CT}}_{\mathrm{reg}}\left(p,k\right)‐{\mathrm{sCT}}_{\theta }\left(p,k\right)\right|$$

**FIGURE 3:**
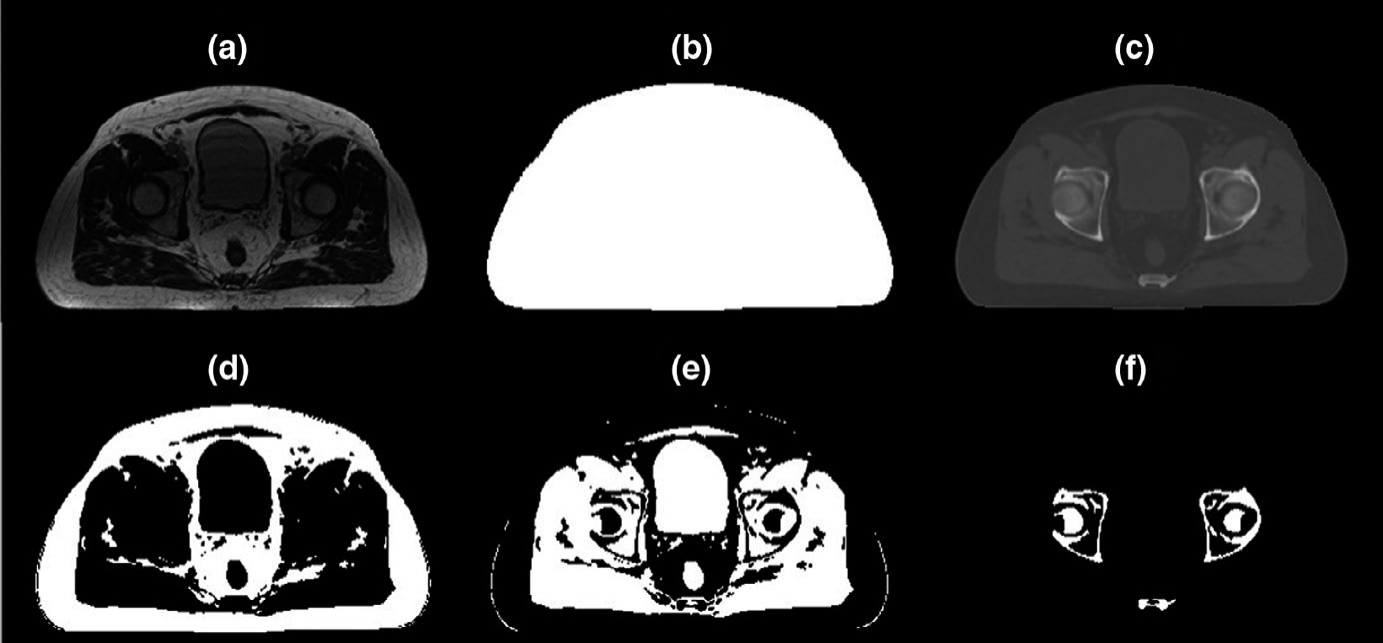
Classification maps generated for a coregistered MRI‐CT atlas. (a) original TRUFI MRI, (b) body contour, obtained using ViewRay treatment planning system, (c) deformed planning CT, (d) fat, (e) muscle, and (f) bone classification maps

In the above, L represents the loss function, and *N* and *B* are the total number of image pixels (224 ∗ 320) and mini‐batch size used in training the model, respectively. One major limitation of using this loss function is that it is heavily weighted by the background, fat and muscle tissues and neglects the impact of bony areas as less bone voxels exist in the image compared to other tissues. Since bony regions have higher Hounsfield Unit (HU), inaccuracy of HU assignment in the final model could lead to a larger dosimetric error for the nearby target and OARs. Hence, we modified the loss function in a way that all tissues contributed equally during the training. To do so, body contour obtained from the treatment planning system was used to extract surface body in the coregistered CT (CT_reg_). Using thresholding (H < −250), air voxels in body contour were also removed from the image. Fuzzy‐c‐means (FCM) clustering was then utilized to classify the remaining voxels into fat, muscle, and bone. Doing so generates four classification maps corresponding to body surface, fat, muscle and bony tissues for each CT_reg_ in our atlas. Each classification map, ${\mathrm{MAP}}_{T},T\in \left\{body,\;fat,\;muscle\;and\;bone\right\}$, is a binary image wherein tissue voxels are set to one and the rest to zero, Figure 3. Using classification map, contributing factor (CF) corresponding to each tissue type was then calculated as follows:

(2) $${\mathrm{CF}}_{T}=\frac{N_{T}}{N_{\mathrm{Total}}},\;\;\;\;T\in \left\{\mathrm{body},\mathrm{fat},\mathrm{muscle},\mathrm{and}\;\mathrm{bone}\right\}$$

where *N*_T_ denotes the number of bright voxels in the corresponding classification map and *N_Total_* represents the total number of voxels in CT_reg_. This procedure was performed for all patients in the training dataset to achieve a set of contributing factors for each patient. The enhancing factor (EF) corresponding to each tissue was then obtained as follows:

(3) $${\mathrm{EF}}_{T}=\frac{n}{\sum_{i=1}^{n}{\mathrm{CF}}_{T}\left(i\right)}\;,T\in \left\{\mathrm{body},\mathrm{fat},\mathrm{muscle},\mathrm{and}\;\mathrm{bone}\right\}$$

where *n* denotes the number of patients in the training dataset. Using the classification map related to each tissue type and the corresponding enhancing factor, the equalization map was then calculated and incorporated into the loss function:

$${\mathrm{MAP}}_{\mathrm{eq}}=J_{N}+\sum_{T}{\mathrm{EF}}_{T}∙{\mathrm{MAP}}_{T},\;T\in \left\{\mathrm{body},\mathrm{fat},\mathrm{muscle},\mathrm{and}\;\mathrm{bone}\right\}$$

(4) $$L_{\mathrm{eq}}\left(\theta \right)=\frac{1}{N*B}\sum_{p=1}^{N}\sum_{k=1}^{B}{\mathrm{MAP}}_{\mathrm{eq}}\left(p,k\right)∙\left|{\mathrm{CT}}_{\mathrm{reg}}\left(p,k\right)‐{\mathrm{sCT}}_{\theta }\left(p,k\right)\right|$$

where *J*_N_ is a matrix whose every element is one and used as the weighting factor for background air voxels and guarantees that each voxel has a contribution in loss function calculation.

#### 2.3.4 Data augmentation

Data augmentation is used frequently in deep learning to increase the amount of data by adding slightly modified copies of already existing dataset^24^ and acts as a regularizer to reduce overfitting of the model during training. Geometric transformation, flipping, rotation, etc. are examples of data augmentation techniques widely used in deep learning. Flipping with respect to median plane and rotation with the range of [−10° 10°] were used to augment the data in this work. As all images were centered before augmentation, no geometric translation was used to augment the data. Unlike traditional data augmentation in which variations of data are added to the initial dataset, in this work, each instance of original data was randomly flipped and rotated and solely used for each round of data propagation through the networks (epoch). As a result, the size of the dataset remained constant, but a new variation of data was used in training for each epoch. This procedure reduces the training time and could eliminate the potential bias certain data points may induce in training presumably resulting to a more robust model.

#### 2.3.5 Training and evaluation details

Three‐fold cross validation was used in this work to evaluate the performance of our proposed model and each set of model's parameters. The dataset was randomly divided into three groups each consisted of ten patients. Each group of patients was once used as a testing dataset while the remaining groups were used for training.

Each MRI‐CT pair was centered, and background margin was truncated so that each pair has a matrix size of 224 × 320. A three‐channel input image was then constructed for each MRI slice and used as input of our deep learning model. Performing this for all patients generated 3258 (ranged from 143 to 186, and median of 161 slices per patient), 3318 (ranged from 150 to 186, and median of 163 slices per patient), and 3218 (ranged from 143 to 176, and median of 161 slices per patient) three‐channel input images for training dataset in group one, two and three, respectively. The co‐registered CT corresponding to each input image was used to calculate the loss function and update the model's parameters in each training step. The same procedure was also applied to create the testing dataset for each group. Our CNN model used a batch size of 4 for input image with Adam optimizer ($\beta_{1}=0.975$, $\beta_{2}=0.999$) and learning rate (α) of 0.001. For each set of training variables and dataset, the model was trained for up to 100 epochs. After completion of each epoch, model's parameters were saved and used to generate synthetic CT for all patients in the testing dataset. For each patient, mean absolute error (MAE) between synthetic and planning CTs were calculated for body contour, fat, muscle, and bone tissues using the corresponding classification map.

To study the relationship between the number of patients and the accuracy of the training procedure, our model was trained and tested using 1, 2, 5 10, 15 and 20 patients, separately for each group of patients. We trained the model using, randomly selected, 4 one‐patient, 4 two‐patient, 4 five‐patient, 2 ten‐patient, 1 fifteen‐patient and 1 twenty‐patient datasets, respectively. If a patient was used in 1‐patient dataset, we made sure that it was also used in 2‐patient dataset and so on. The results were then averaged in each category and also for all three groups of patients. To quantify and compare the impact of each preprocessing step and model's parameter, we also trained and tested the model by changing only one variable at a time and then used paired student *t* test to find any significant differences between the two set of variables. As a result, the model was trained with and without histogram standardization, data augmentation and equalized loss function. Furthermore, in order to compare our proposed data augmentation with traditional approach, new datasets comprising of the original dataset and 5 randomly‐generated variations of the original data was created for each group of patients. As a result, we constructed three sets of training dataset comprising of 19548, 19908, and 19308 input images, respectively which were then used to train and test the model. We also replaced the mean absolute error (MAE) in the loss function, Equation (4), with mean square error (MSE) and then trained and tested the model to compare the role of MAE and MSE in training. All training procedures were done in MATLAB (Version 9.7, R2020a, MathWorks, Inc.) with GPU enabled (NVIDIA^®^ GeForce^®^ GTX 1660 Ti 6GB GDDR6) deep learning toolbox.

## 3 RESULTS

**FIGURE 4:**
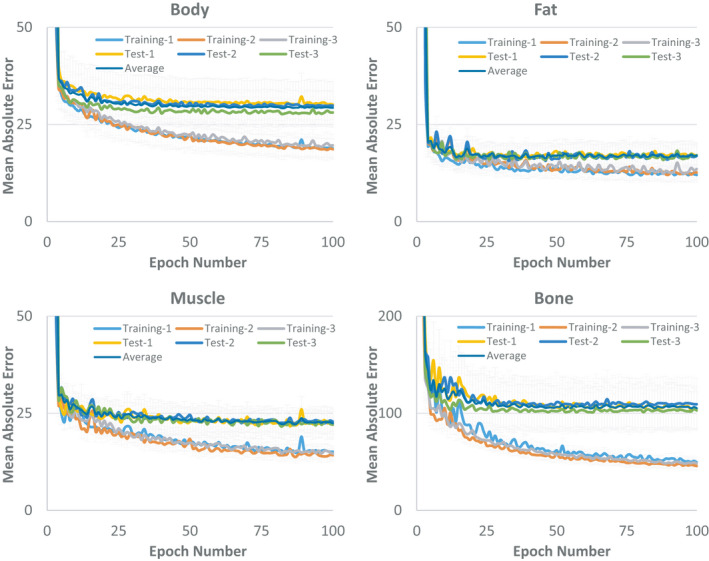
Changes in mean absolute error (MAE) between synthetic and deformed planning CT in body contour, fat, muscle, and bony tissues as the training time increases. Each training was performed using twenty patients and up to 100 epochs. Each data point represents an average of MAE over the entire training and testing dataset and the error bar denotes the standard deviation

Figure 4a–4d shows how mean absolute error (MAE) between synthetic and deformed planning CT changed in various tissues as training time, epoch number, increased for all three groups of patients. Each data point represents an average of MAE over the entire training and testing dataset and the error bar denotes the standard deviation. Each training was performed using twenty patients and model's parameters were saved after each epoch to generate synthetic CT for both training and testing patients. As shown, increasing the training time reduces the MAE in the training dataset as loss function decreases, but does not necessary generate a better model as MAE in the testing dataset remains steady after a certain time. In other words, each dataset might have certain learning capacity achieved after a certain period and increasing the training time beyond this point does not necessarily improve the learning process. It's worthwhile to note that the lower curves in Figure 4a–4c also represent the training errors over fat, muscle, bone, and body contours averaged for all slices and all patients.

**FIGURE 5:**
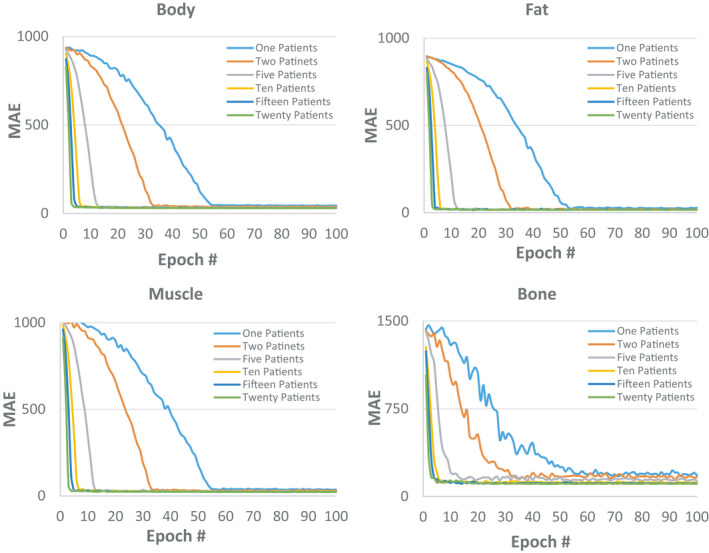
A typical example of changes in time when a learning capacity of a dataset was reached as the number of patients in training increased. Each point on a curve represents the average of MAE between synthetic and deformed planning CT calculated using the testing dataset. The results were achieved using the second group of 20 patients

**FIGURE 6:**
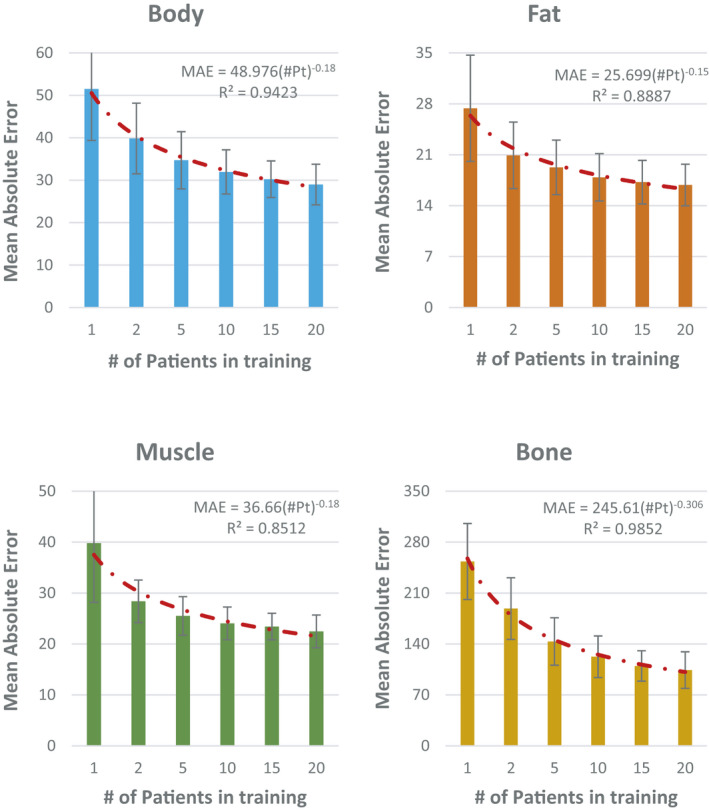
Changes in mean absolute error (MAE) between synthetic and deformed planning CT in body contour, fat, muscle, and bony tissues as number of training patients increased. Each data point represents an average of MAE over the testing patients in all three groups considering all variations of training samples explained in Section 2.3.5. Error bar denotes standard deviation. Results were achieved by averaging the MAE over epochs 71 through 75

Figure 5a–5d shows a typical example of changes in time when a learning capacity of a dataset was reached as the number of patients in training increased. Each point on a curve represents the average of MAE between synthetic and deformed planning CT calculated using the testing dataset. The results were achieved using the second group of patients. Figure 5 implies that with less patients in a dataset, more epochs are needed to reach the learning capacity of that dataset. Our complete results indicate that after almost 60 epochs, the learning capacity of all datasets were reached. Hence, to quantify the impact of the number of patients in training, we trained each dataset for up to 75 epochs and then took the average of MAE over the last 5 epochs (71–75) as an indicator of the learning capacity of that dataset. Figure 6a–6d shows how MAE between synthetic and deformed planning CT changed in various tissues as number of patients in training increased. Each data point represents an average of MAE over the testing patients in all three groups considering all variations of training samples explained in section 2.C.5. Error bar denotes standard deviation. Figure 6 confirms that increasing the number of training patients improves the learning capacity of a dataset but also denotes that the MAE and the number of patients in training are non‐linearly corelated with the largest slope observed in bony tissue. For instance, Figure 6a shows that using only 1 patient in training, we could achieve a MAE of about 51.51 ± 12.14 Hounsfield Unit (HU) for the entire body contour. This value reduced to 28.98 ± 4.79 HU when the number of patients reached 20. However, it shows that to reduce this value by 50% (14 HU), we may need to include more than 1000 patients in the training dataset.

**FIGURE 7:**
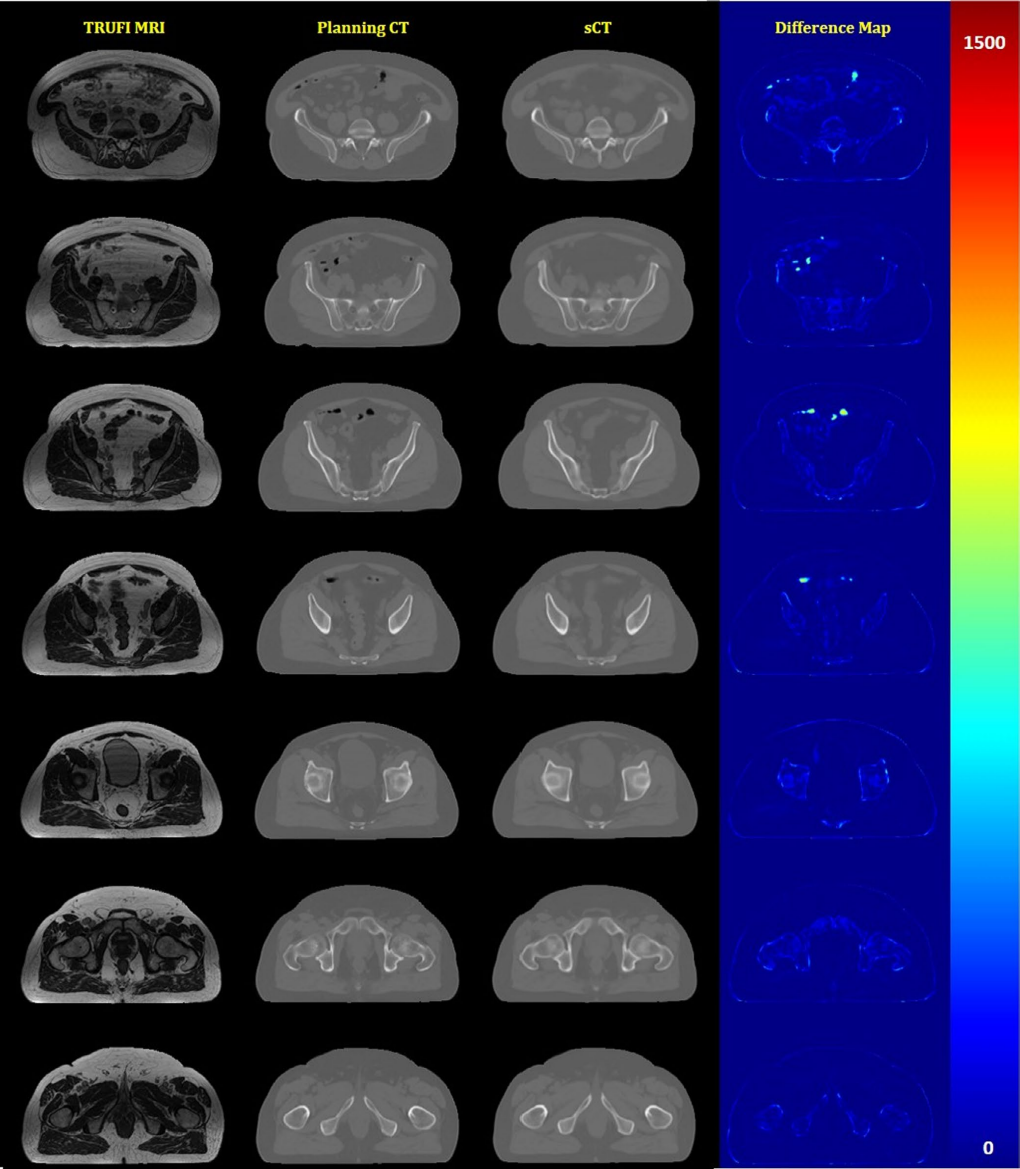
Example of sCT generated using our proposed deep learning model

Table 1 shows how MAE between synthetic and deformed planning CT changed when different variables and input images were used in training. Each table row represents the average of MAE ±standard deviation in the last 5 (46–50) epochs calculated using twenty patients in training and when training continued for up to 50 epochs. As indicated in Figure 5, 50 epochs could be more than enough for training our model with a dataset comprising of twenty patients. In overall, Table 1 shows that changes in training variables mainly affected MAE in bony regions and had little effect on fat and muscle tissues. Among all training variables, weighted loss function had the highest impact and lowered the MAE from 129.25 ± 30.52 to 105.90 ± 22.80 HU in bone voxels (*p* < 10^−13^). The first three rows in Table 1 show that both traditional data augmentation and one used in this work generated a better MAE in bone tissue. However, no significant difference was observed between the two augmentation techniques (*p* < 0.4). It is worthwhile to note that the size of the training data in the traditional data augmentation is usually much larger than the original dataset depending on the amount of new data added to the original samples. In this case, the size of the augmented dataset was six times larger than the original dataset requiring longer training time proportionately. Interestingly, the results show that standardization did not help to achieve better results and the training using original images provided a significantly better MAE over bone tissue (*p* < 0.0004). We finally found out that in overall MAE may provide a better model when used as the loss function compared to MSE as the average of MAE over the fat (*p* < 2 × 10^−8^) and muscle (*p* < 3 × 10^−9^) tissues increased significantly with no major improvement over the bone tissue (*p* < 0.055). An example of synthetic CT generated using our proposed method is shown in Figure 7.

**TABLE 1:** Mean absolute error (MAE) between synthetic and deformed planning CT calculated in body, fat, muscle, and bone tissues using models trained with different image inputs and variables

	Whole body	Fat	Muscle	Bone
Main method	29.68 ± 4.41	16.34 ± 2.67	23.36 ± 2.85	105.90 ± 22.80
Traditional augmentation	29.09 ± 4.58	16.32 ± 2.90	22.27 ± 2.70	106.48 ± 22.45
No augmentation	30.17 ± 4.92	16.17 ± 3.20	23.21 ± 2.38	115.89 ± 23.48
Standardized MRI	30.17 ± 4.57	16.61 ± 3.25	23.95 ± 2.81	109.99 ± 22.65
Unweighted loss function	30.62 ± 4.95	17.40 ± 2.85	22.88 ± 2.79	129.25 ± 30.52
Mean square error	31.06 ± 4.13	18.07 ± 2.64	25.35 ± 2.77	105.06 ± 22.18

Note

The result is achieved using three‐fold cross‐validation where 20 patients were used in training and 10 for testing.

## 4 DISCUSSION

A U‐NET‐based deep learning model has been developed and tested to estimate electron density map from TRUFI MRI for MR‐only radiotherapy of prostate cancer patients treated with 0.35T MRI linear accelerator. In our MRgRT workflow, patients are simulated with both CT and MRI. MRI is used for target delineation and OARs contouring and CT is used for dose calculation. MRI and CT simulation images were initially pre‐processed, co‐registered and then used for training and testing of our deep leaning model. In this work, we've also proposed a new cost function to improve the accuracy of HU assignment in bony regions. We also explored the advantage of a data augmentation approach where instead of the original data, only variations of the dataset were used in training. The impact of training time, image standardization was also quantified by measuring the mean absolute value (MAE) between synthetic and planning CT in various tissue types.

Our experimental results show that with twenty patients in training, our deep learning model has the potential to generate synthetic CT with a MAE of 29.68 ± 4.41, and 105.90 ± 22.80 HU over the entire body, and bony regions compared to that of 47 ± 5.88 and 172 ± 9HU achieved for MRCAT^5^, ^7^ (the first commercial synthetic CT proposed for mDixon sequence) and 54.31 ± 11.87 and 224.335 ± 35.45 HU achieved for one of the most recent deep learning approaches developed using 36 patients for 0.35T MRI‐Linac.^10^ To improve the performance of our deep learning model on high density regions, we introduced a new cost function that equalized the impact of all tissue voxels in calculation of the loss function. As background air, fat and muscle comprise more voxels than bony regions, they may result to neglect high density voxels in loss function calculation. The new loss function calculation improved the MAE by more than ~18% in high density areas. One other important factor is that we used the first order distance (MAE) between the synthetic and deformed planning CT to calculate the loss function. In fact, replacing MAE with MSE may excessively enhance the difference in bony regions and neglect the difference in fat and muscle tissue leading to higher MAE over the fat and muscle tissues, respectively (Table 1).

Our results confirm that increasing the number of training patients plays a vital role to improve the accuracy of a deep learning model with the greatest impact on bony tissues. As expected, the number of training patients and MAE are non‐linearly correlated. This means that adding patients to a small dataset helps generating a better model significantly but when the number of patients is large, adding few patients may only slightly improve the training process. For instance, Figure 6d shows that with only 1 patient, a MAE of ~253.36 ± 52.16 (HU) was achieved in bone voxels. Increasing the number of patients to 10 improved the MAE by ~50% resulted to a MAE of ~122.43 ± 28.57 HU for high density regions. Increasing 10 more patients in training only improved MAE by ~15% (104.14 ± 25.16). Figure 6d shows that to improve MAE in bone voxels by another ~50%, we may need to have more than 200 patients in training. Similarly, Figure 6a shows that with 20 patients, an overall MAE of ~28.98 ± 4.79 HU is achievable. However, to reduce this value by nearly 50%, we may need to include more than 1000 patients in the training dataset. This implies that there should be a trade‐off between the desirable MAE and the size of the training dataset.

In this work, we used a data augmentation technique that included a new variation of the original data for each round of training. In this way, it is guaranteed that no input data is repeatedly used during the training. This could eliminate the potential bias a specific data point may have on training and generate a robust model. Also, as the size of the dataset does not increase in this way, in contrast to traditional data augmentation where variations of data are added to the dataset, the whole training process is faster. This is especially important if we need to include hundreds of patients in training. Table 1 shows that both ways of data augmentation could achieve similar results for fat, muscle, and bone tissues.

One of our major finding in this work was that increasing the training time does not necessarily produce a better predictive model. In other word, each dataset might have certain training capacity which is reached after a certain period and beyond this point no major improvement can be achieved. For instance, in this work, we trained the model for up to 100 epochs for each set of training inputs and variables and also for different number of patients in the dataset. Figure 4 shows that, with 20 patients in training, we might have needed only 30 to 40 epochs to train the model. Hence, although training is done offline, it can be shortened if needed. In fact, Figure 4 shows that after 25 epochs, the model can easily learn how to map the fat and muscle tissues, but our numerical experiments (not shown here) revealed that we need a bit more epochs to make sure that mapping of the bony voxels are also properly accomplished. Also, Figure 5 shows that we need more epochs in training when the number of patients in the training dataset decreases. This is mainly because of having lower number of iterations per epoch when the lower number of patients exist in the dataset. It is also worthwhile to note that increasing the training time may also achieve a model with training parameters specific to the training dataset leading to an overfitted model. Interestingly, Table 1 also shows that image standardization does not necessarily help to achieve a better model hence is not needed for routine clinical use as original image data can generate an appropriate electron density map. This will eliminate the complexity of the synthetic CT generation process and facilitate the clinical workflow. However, our standardization needs manual segmentation of fat and muscle tissues that is one of the limitations of our approach.

Looking at the difference maps between the synthetic and deformed planning CT in Figure 7 shows that air voxels in the image are the areas with the largest MAE in our model. However, since the location of air bubbles in the pelvis regions and especially in the rectum could vary and may not be even the same in planning CT and MRI, we may need to explore other techniques to properly map the electron density value to these regions. One easy solution to this problem is to manually contour the air voxels in MRI and assign them a bulk density value. Another interesting solution is to develop an independent model to segment the air voxels in the image. As the synthetic CT could also provide the location of bone tissues in the image, it may also be used as guidance for proper classification of low intensity voxels into air or bone tissues. This will be part of our future works to prepare a clinically usable product.

In close, we have developed and investigated the feasibility of a deep learning model to generate synthetic CT for therapeutic MRI images. In our experiment, we used mean absolute error to measure the goodness of the model and found that the number of training patients is crucial in the accuracy of a model and there should be a tradeoff between the desired accuracy and the number of patients needed. In our future work, we aim to perform a dosimetric study for clinical evaluation of our proposed model and find out how many patients will be enough for a safe usage of our model in routine clinical workflow. One consideration that should also be thoroughly studied in dosimetric assessment of synthetic CT product is to develop and stablish an effective quality assurance program for routine clinical usage of such products. In fact, it is quite probable that for some patients, the knowledge from the dataset will produce erroneous results leading to severe dosimetric errors. Hence, it is essential to have independent tools and protocol to detect such discrepancy and prevent their adverse effects. Esposito et al.^25^ have recently reviewed some of the potential in vivo dosimetric tools like point dosimeters that can be used for such purposes. Farjam et al.^7^ have also shown that replacing the entire electron density map with water may also be a good estimation for pelvis anatomy and can be used as an independent electron density map for the purpose of dosimetric comparison. Hence, in the future, we also plan to work on developing appropriate quality assurance program for the purpose of using synthetic CT for MR‐only radiotherapy of prostate cancer patients.

## 5 CONCLUSION

A deep learning model has been developed and tested in this work to generate synthetic CT from 0.35 T TRUFI MRI for MR‐only radiotherapy of prostate cancer patient. A new cost function equalizing the contribution of different tissue types was found useful to enhance the performance of our U‐NET based model in estimating Hounsfield Unit in bony regions. As expected, we observed that the number of training patients and mean absolute error between synthetic and planning CT are nonlinearly correlated. Interestingly, we found that increasing the training time and MRI histogram standardization do not play a significant role in the goodness of the final model. Dosimetric evaluation of the proposed model using a large and independent dataset warrants the validity of the proposed model.

## CONFLICT OF INTEREST

The authors have no relevant conflicts of interest to disclose.

## AUTHOR CONTRIBUTIONS

Reza Farjam: Study design, data collection and analysis, manuscript drafting and approving; Himanshu Nagar: Data acquisition, manuscript revision and approving; Xi Kathy Zhou: Study design, manuscript revision and approving; David Ouellette: Data analysis, manuscript revision and approving; Silvia Chiara Formenti: Data acquisition, manuscript revision and approving; J Keith Dewyngaert: Study design, manuscript revision and approving.
